# Supplementary material for: The Small RNA Universe of Capitella teleta
Source: Front Mol Biosci. 2022 Feb 25;9:802814. doi: 10.3389/fmolb.2022.802814 (PMC8915122; doi:10.3389/fmolb.2022.802814)
Supplement: Supplementary file 1 [file DataSheet1.ZIP › Supplement/known/CAPTEscaffold_14_1882.pdf]

Provisional ID	:	CAPTEscaffold_14_1882
Score total	:	293877.7
Score for star read(s)	:	3.9
Score for read counts	:	293871.5
Score for mfe	:	1.5
Score for randfold	:	-2.2
Score for cons. seed	:	3
Total read count	:	576428
Mature read count	:	575826
Loop read count	:	0
Star read count	:	602

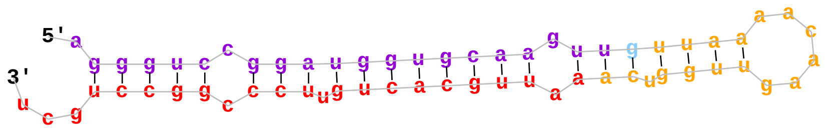

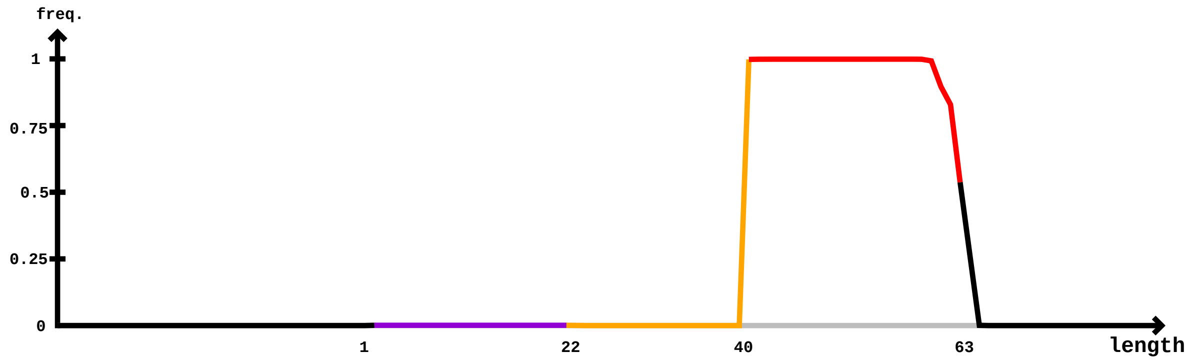

Star　　　　　　　　　　Mature

```
5'- gagugaccaccgcauggcgucguugaaucggaggguccggauggugcaaguuguuaaaacaaguuggucaaauugcacuguucccggccugcugaucucacucggccgauaac  -3'  obs
    gagugaccaccgcauggcgucguugaaucggaggguccggauggugcaaguuguuaaaacaaguuggucaaauugcacuguucccggccugcugaucucacucggccgauaac       exp
    gagugaccaccgcauggcgucguugaaucggaggguccggauggugcaaguuguuaaaacaaguuggucaaauugcacuguucccggccugcugaucucacucggccgauaac       known
    ..(((.....)))((.(.((((.((((((((.(((((.((((((((((.(((((((......)))).))).))))))))).))).))))).))))).)))..))))).))...     reads   mm   sample
    ...........gcauggcgucguugaaucgg..................................................................................     2       0    seq
    ............cauggcgucguugaaucgg..................................................................................     1       0    seq
    ...............................aggguccggauggugcaagu..............................................................     13      0    seq
    ...............................aggguAcggauggugcaaguu.............................................................     1       1    seq
    ...............................aggguccggauAgugcaaguu.............................................................     1       1    seq
    ...............................aggguccggauggugcaaguu.............................................................     288     0    seq
    ...............................aggguccggauggAgcaaguu.............................................................     1       1    seq
    ...............................Gggguccggauggugcaaguu.............................................................     7       1    seq
    ...............................aggguccggauggugcaaguuA............................................................     3       1    seq
    ...............................aggguAcggauggugcaaguug............................................................     1       1    seq
    ...............................aggguccggauAgugcaaguug............................................................     1       1    seq
    ...............................aggguccggauggugcaaguug............................................................     272     0    seq
    ...............................aggguccAgauggugcaaguug............................................................     1       1    seq
    ...............................Gggguccggauggugcaaguug............................................................     3       1    seq
    ...............................aggguccggauggugcaaguugu...........................................................     8       0    seq
    ...............................aggguccggauggugcaaguugA...........................................................     2       1    seq
    .....................................................................Caauugcacuguucccggccu.......................     8       1    seq
    .....................................................................Caauugcacuguucccggccugc.....................     3       1    seq
    .....................................................................Caauugcacuguucccggccugcu....................     8       1    seq
    ......................................................................aauugcacuguucccggc.........................     180     0    seq
    ......................................................................aauugcacuguucccggA.........................     4       1    seq
    ......................................................................aauugcacuguucccgUcc........................     1       1    seq
    ......................................................................Gauugcacuguucccggcc........................     1       1    seq
    ......................................................................Nauugcacuguucccggcc........................     1       1    seq
    ......................................................................aauugcacugAucccggcc........................     1       1    seq
    ......................................................................aauugcacuguucccgAcc........................     1       1    seq
    ......................................................................aauugcacAguucccggcc........................     1       1    seq
    ......................................................................aauugcacuguucAcggcc........................     1       1    seq
    ......................................................................aauugcacuguucccggcc........................     3524    0    seq
    ......................................................................aauugcacuguAcccggcc........................     2       1    seq
    ......................................................................aauugcacuguuccUggcc........................     2       1    seq
    ......................................................................aauugcUcuguucccggcc........................     1       1    seq
```

Star **Mature**

gagugaccaccgcauggcgucguugaaucggagggguccggauggugcaaguuguuaaaacaaguuggucaaauugcacuguucccggccugcugaucucacucggccgaauaac

....aauugcCcuguucccggcc.........................	1	1	seq
....aauuCcacuguucccggcc.........................	2	1	seq
....aauugcacuguucccggcA.........................	1	1	seq
....aauugcaUuguucccggcc.........................	1	1	seq
....aauugcacuguucUcggcc.........................	1	1	seq
....aauCgcacuguucccggcc.........................	1	1	seq
....aauAgcacuguucccggcc.........................	3	1	seq
....aauuUcacuguucccggcc.........................	2	1	seq
....aauugcacuguucccggGc.........................	1	1	seq
....aauugAacuguucccggcc.........................	3	1	seq
....aauugcacuguuccAggcc.........................	1	1	seq
....aauugcacuguuAccggcc.........................	1	1	seq
....aauugcaAuguucccggcc.........................	2	1	seq
....aauugcacuUuucccggcc.........................	1	1	seq
....aauugcacuguucccUgccu........................	4	1	seq
....aauuAcacuguucccggccu........................	6	1	seq
....aauuCcacuguucccggccu........................	6	1	seq
....aNuugcacuguucccggccu........................	2	1	seq
....aauugcacuguucGcggccu........................	4	1	seq
....aauugcacAguucccggccu........................	12	1	seq
....aauugcacuNuucccggccu........................	1	1	seq
....aauugcacuguuccAggccu........................	12	1	seq
....aauugcacuguucccgNccu........................	1	1	seq
....aauugcacuguucccgAccu........................	7	1	seq
....aauugcaAuguucccggccu........................	9	1	seq
....Gauugcacuguucccggccu........................	39	1	seq
....aauugcacCguucccggccu........................	5	1	seq
....aaCugcacuguucccggccu........................	2	1	seq
....aaGugcacuguucccggccu........................	1	1	seq
....aauugcacuguucccgCccu........................	2	1	seq
....aauugcacuguucccggcGu........................	1	1	seq
....aaAugcacuguucccggccu........................	48	1	seq
....aauugcUcuguucccggccu........................	9	1	seq
....aauugcGcuguucccggccu........................	25	1	seq
....aauugcacuguuAccggccu........................	33	1	seq
....aauugcacuguucccggAcu........................	12	1	seq
....aGuugcacuguucccggccu........................	12	1	seq
....aauugcacGguucccggccu........................	4	1	seq
....aauugcacuguucccggccG........................	322	1	seq
....aauAgcacuguucccggccu........................	20	1	seq
....aauugUacuguucccggccu........................	4	1	seq
....aauugAacuguucccggccu........................	39	1	seq
....aauugcacugAucccggccu........................	24	1	seq
....aauugcacuguucccggcAu........................	9	1	seq
....aauugcacuguucccggUcu........................	5	1	seq
....aauugcacuguuGccggccu........................	2	1	seq
....aauugcacuguucccggccA........................	88	1	seq
....aauugcacuAuucccggccu........................	5	1	seq
....aauugcacuguuUccggccu........................	12	1	seq
....aauugcacuguuccUggccu........................	33	1	seq
....aauugcacuUuucccggccu........................	14	1	seq
....Uauugcacuguucccggccu........................	2	1	seq
....aauugcCcuguucccggccu........................	2	1	seq
....aauugcacuguucccgUccu........................	1	1	seq
....aUuugcacuguucccggccu........................	6	1	seq
....aauugcacugCucccggccu........................	2	1	seq
....aauugcaUuguucccggccu........................	8	1	seq
....aauugGacuguucccggccu........................	4	1	seq
....aauugcacuguucccggccC........................	7	1	seq
....aauugcacuguucAcggccu........................	19	1	seq
....aauugcacuguucccCgccu........................	2	1	seq
....aauugcacuguucccggccu........................	54477	0	seq
....aauuUcacuguucccggccu........................	8	1	seq
....aauugcacuguucccAgccu........................	29	1	seq
....aauugcacuguuccGggccu........................	7	1	seq
....aauugcacuguAcccggccu........................	20	1	seq
....aauugcacuguucccggGcu........................	5	1	seq
....aauugcacugGucccggccu........................	8	1	seq
....aauugcacuguucUcggccu........................	31	1	seq
....aauugcacuguGcccggccu........................	1	1	seq

Star Mature

gagugaccaccgcauggcgucguugaaucggaggguccggauggugcaaguuguuaaaacaaguuggucaaauugcacuguucccggccugcugaucucacucggccgauaac			
....aauugcacuguucccggcUu.......................	11	1	seq
....Nauugcacuguucccggccu.......................	17	1	seq
....aauugcacuguCcccggccu.......................	11	1	seq
....aauCgcacuguucccggccu.......................	8	1	seq
....aauugcacuCuucccggccu.......................	3	1	seq
....aauugcacuguucccggcAug......................	6	1	seq
....aauugcacuguucccggccuC......................	7	1	seq
....aauugcaUuguucccggccug......................	2	1	seq
....aauugcacuguucccgCccug......................	3	1	seq
....aauugcacuguucccggcGug......................	6	1	seq
....aauugcacAguucccggccug......................	5	1	seq
....aauugcacuguucGcggccug......................	4	1	seq
....aauugcacuAuucccggccug......................	1	1	seq
....aauugcacuguucccggccuA......................	10	1	seq
....Gauugcacuguucccggccug......................	48	1	seq
....aauugcUcuguucccggccug......................	4	1	seq
....aauugcacuguGcccggccug......................	1	1	seq
....aGuugcacuguucccggccug......................	1	1	seq
....aauugAacuguucccggccug......................	26	1	seq
....Nauugcacuguucccggccug......................	2	1	seq
....aauugcacuguucccUgccug......................	4	1	seq
....aauugcacuguucccggccuU......................	210	1	seq
....Uauugcacuguucccggccug......................	1	1	seq
....aauugcacuguucccggccGg......................	334	1	seq
....aauugcacuguAcccggccug......................	10	1	seq
....aauugcacuguucccggccAg......................	4	1	seq
....aauugcacugCucccggccug......................	2	1	seq
....aUuugcacuguucccggccug......................	3	1	seq
....aauugcacuguucccggccug......................	37777	0	seq
....aaCugcacuguucccggccug......................	2	1	seq
....aauugcacugAucccggccug......................	16	1	seq
....aauuAcacuguucccggccug......................	8	1	seq
....aauugcacuguucccggUcug......................	6	1	seq
....aauugcacuguucccggAcug......................	9	1	seq
....aauugcacuguucccggcUug......................	8	1	seq
....aauugcacuguucccgUccug......................	3	1	seq
....aauugcacuguucAcggccug......................	17	1	seq
....aauugcacuCuucccggccug......................	2	1	seq
....Cauugcacuguucccggccug......................	1	1	seq
....aauugcacuguuUccggccug......................	4	1	seq
....aauugcaAuguucccggccug......................	8	1	seq
....aauugcacuguuccGggccug......................	4	1	seq
....aauugcacuguuAccggccug......................	29	1	seq
....aauugcacuguucUcggccug......................	21	1	seq
....aauugUacuguucccggccug......................	2	1	seq
....aauugcacuguucccCgccug......................	1	1	seq
....aauugcacuguucccggGcug......................	7	1	seq
....aauuUcacuguucccggccug......................	6	1	seq
....aauugcGcuguucccggccug......................	16	1	seq
....aauugGacuguucccggccug......................	4	1	seq
....aauugcacuguucccggccCg......................	3	1	seq
....aauugcacuguuccAggccug......................	18	1	seq
....aauuCcacuguucccggccug......................	1	1	seq
....aauCgcacuguucccggccug......................	6	1	seq
....aauAgcacuguucccggccug......................	15	1	seq
....aauugcacuguCcccggccug......................	4	1	seq
....aaAugcacuguucccggccug......................	37	1	seq
....aauugcacGguucccggccug......................	5	1	seq
....aauugcacuguucccAgccug......................	14	1	seq
....aauugcacugGucccggccug......................	13	1	seq
....aauugcacCguucccggccug......................	5	1	seq
....aauugcacuguuGccggccug......................	1	1	seq
....aauugcaGuguucccggccug......................	1	1	seq
....aauugcacuguuccUggccug......................	29	1	seq
....aNuugcacuguucccggccug......................	1	1	seq
....aauugcacuUuucccggccug......................	34	1	seq
....aauugcacuguucccgAccug......................	5	1	seq
....Cauugcacuguucccggccugc.....................	2	1	seq
....aauugcaAuguucccggccugc.....................	28	1	seq
....aauugcacuguucccggcGugc.....................	8	1	seq

```
                               Star                                   Mature
gagugaccaccgcauggcgucguugaaucggaggguccggauggugcaaguuguuaaaacaaguuggucaaauugcacuguucccggccugcugaucucacucggccgauaac
......................................................................aauugcacuguucccggccugc.....................   159619   0   seq
......................................................................aauAgcacuguucccggccugc.....................   71       1   seq
......................................................................aaNugcacuguucccggccugc.....................   1        1   seq
......................................................................aauugcacugCucccggccugc.....................   32       1   seq
......................................................................aauugcacuguuccAggccugc.....................   53       1   seq
......................................................................aauugcacugAucccggccugc.....................   57       1   seq
......................................................................aauugcacuguAcccggccugc.....................   61       1   seq
......................................................................aauugcacuguuccUggccugc.....................   100      1   seq
......................................................................aauugcacuNuucccggccugc.....................   1        1   seq
......................................................................aauugcaUuguucccggccugc.....................   9        1   seq
......................................................................aauugcacuguucccggccCgc.....................   16       1   seq
......................................................................aauugcaNuguucccggccugc.....................   1        1   seq
......................................................................aauugcacuguucccggccAgc.....................   20       1   seq
......................................................................aGuugcacuguucccggccugc.....................   14       1   seq
......................................................................Nauugcacuguucccggccugc.....................   44       1   seq
......................................................................aaGugcacuguucccggccugc.....................   1        1   seq
......................................................................aauugcacuguucccggccuCc.....................   8        1   seq
......................................................................aauugcacuAuucccggccugc.....................   20       1   seq
......................................................................aauugcGcuguucccggccugc.....................   52       1   seq
......................................................................aauugcacuUuucccggccugc.....................   353      1   seq
......................................................................aauugcacuguucccAgccugc.....................   58       1   seq
......................................................................aauugcacuguucccggAcugc.....................   32       1   seq
......................................................................aauugcacGguucccggccugc.....................   7        1   seq
......................................................................aauugcUcuguucccggccugc.....................   22       1   seq
......................................................................aCuugcacuguucccggccugc.....................   2        1   seq
......................................................................aauugcacuguCccggccugc......................   20       1   seq
......................................................................aauugcacuguucAcggccugc.....................   82       1   seq
......................................................................aauugcacuguucccggcUugc.....................   32       1   seq
......................................................................aauugcacuguucccgUccugc.....................   8        1   seq
......................................................................aauugcacNguucccggccugc.....................   2        1   seq
......................................................................aauugAacuguucccggccugc.....................   97       1   seq
......................................................................aauugcacuguucccUgccugc.....................   6        1   seq
......................................................................aauugcacuguucccggUcugc.....................   15       1   seq
......................................................................aauugcacuguucUcggccugc.....................   109      1   seq
......................................................................aauGgcacuguucccggccugc.....................   2        1   seq
......................................................................aauugcacuguucccggccuUc.....................   9        1   seq
......................................................................aauugcacuguuGccggccugc.....................   14       1   seq
......................................................................aaAugcacuguucccggccugc.....................   138      1   seq
......................................................................aauugcacuguuUccggccugc.....................   28       1   seq
......................................................................aauuAcacuguucccggccugc.....................   25       1   seq
......................................................................aauugcNcuguucccggccugc.....................   1        1   seq
......................................................................aauugcacuguucccggccugU.....................   5363     1   seq
......................................................................aauugcacuguucGcggccugc.....................   11       1   seq
......................................................................aauugcacuguucccggcAugc.....................   20       1   seq
......................................................................Uauugcacuguucccggccugc.....................   9        1   seq
......................................................................aauugcacuguucccggccGgc.....................   8        1   seq
......................................................................aauugcacuguucccCgccugc.....................   12       1   seq
......................................................................Gauugcacuguucccggccugc.....................   159      1   seq
......................................................................aauugcacuguucccggccugA.....................   414      1   seq
......................................................................aauugGacuguucccggccugc.....................   8        1   seq
......................................................................aauugcacuguucccggccuNc.....................   1        1   seq
......................................................................aauugcacugNucccggccugc.....................   2        1   seq
......................................................................aauuUcacuguucccggccugc.....................   14       1   seq
......................................................................aaCugcacuguucccggccugc.....................   9        1   seq
......................................................................aauugcacuguucccggccugG.....................   71       1   seq
......................................................................aauugcacugGucccggccugc.....................   279      1   seq
......................................................................aauugcacuguGcccggccugc.....................   4        1   seq
......................................................................aauugcacAguucccggccugc.....................   30       1   seq
......................................................................aauugcacuguuccGggccugc.....................   15       1   seq
......................................................................aUuugcacuguucccggccugc.....................   11       1   seq
......................................................................aauugcCcuguucccggccugc.....................   7        1   seq
......................................................................aauugcaGuguucccggccugc.....................   3        1   seq
......................................................................aauugcacuguuAccggccugc.....................   104      1   seq
......................................................................aauugcacCguucccggccugc.....................   12       1   seq
......................................................................aauuCcacuguucccggccugc.....................   3        1   seq
......................................................................aauugcacuguucccggGcugc.....................   16       1   seq
......................................................................aauugcacuguucccgCccugc.....................   7        1   seq
......................................................................aauugcacuguucccggccuAc.....................   17       1   seq
......................................................................aauCgcacuguucccggccugc.....................   21       1   seq
......................................................................aauugNacuguucccggccugc.....................   3        1   seq
```

```
                                         Star                                      Mature
gagugaccaccgcauggcgucguugaaucggaggguccggauggugcaaguuguuaaaacaaguuggucaaauugcacuguucccggccugcugaucucacucggccgauaac
......................................................................aauugcacuCuucccggccugc.....................   18      1   seq
......................................................................aauugUacuguucccggccugc.....................   20      1   seq
......................................................................aauugcacuguucccgAccugc.....................   36      1   seq
......................................................................aauAgcacuguucccggccugcu....................   117     1   seq
......................................................................aauugcacuguucccggccuNcu....................   1       1   seq
......................................................................aUuugcacuguucccggccugcu....................   15      1   seq
......................................................................aauugcCcuguucccggccugcu....................   19      1   seq
......................................................................aauugcacuguucccUgccugcu....................   12      1   seq
......................................................................aauugcacuguuccUggccugcu....................   207     1   seq
......................................................................aauugcaGuguucccggccugcu....................   11      1   seq
......................................................................aauugcacuguGcccggccugcu....................   15      1   seq
......................................................................aauugcacuCuucccggccugcu....................   29      1   seq
......................................................................aauugcacugGucccggccugcu....................   20      1   seq
......................................................................aauuAcacuguucccggccugcu....................   37      1   seq
......................................................................aauugcacuAuucccggccugcu....................   26      1   seq
......................................................................aauugcacuguucccggccGgcu....................   34      1   seq
......................................................................aauugcacuguucccggccuCcu....................   16      1   seq
......................................................................aauuCcacuguucccggccugcu....................   11      1   seq
......................................................................Uauugcacuguucccggccugcu....................   16      1   seq
......................................................................aauugcacuguucccCgccugcu....................   12      1   seq
......................................................................aauugAacuguucccggccugcu....................   142     1   seq
......................................................................aauugcacuguucccggAcugcu....................   57      1   seq
......................................................................aauugcacuguucccggccugcu....................   275248  0   seq
......................................................................aaCugcacuguucccggccugcu....................   17      1   seq
......................................................................aauugcacuguucccggccugcN....................   3       1   seq
......................................................................aauugcacuguucccggccugUu....................   393     1   seq
......................................................................aauugcacuguucccggccugGu....................   13      1   seq
......................................................................aauugcacAguucccggccugcu....................   55      1   seq
......................................................................aauugcacuguucccgUccugcu....................   33      1   seq
......................................................................aauugcacuguuccAggccugcu....................   86      1   seq
......................................................................Nauugcacuguucccggccugcu....................   80      1   seq
......................................................................aaGugcacuguucccggccugcu....................   5       1   seq
......................................................................aauugGacuguucccggccugcu....................   22      1   seq
......................................................................aNuugcacuguucccggccugcu....................   12      1   seq
......................................................................aauugcacuguucccgCccugcu....................   22      1   seq
......................................................................aauugcacuguucccggccuAcu....................   26      1   seq
......................................................................aauugcacuguAcccggccugcu....................   98      1   seq
......................................................................aauugcacCguucccggccugcu....................   34      1   seq
......................................................................aauugcacuguucccggGcugcu....................   19      1   seq
......................................................................aauugcacuguucccggcAugcu....................   28      1   seq
......................................................................aauugcacuUuucccggccugcu....................   40      1   seq
......................................................................aaNugcacuguucccggccugcu....................   1       1   seq
......................................................................aauugcacuguucccggccugcA....................   1046    1   seq
......................................................................aauugcaAuguucccggccugcu....................   40      1   seq
......................................................................aauuUcacuguucccggccugcu....................   20      1   seq
......................................................................aauugcacuguuGccggccugcu....................   22      1   seq
......................................................................aauugcacuguucccggccugcG....................   891     1   seq
......................................................................aauugcacuguuccGggccugcu....................   27      1   seq
......................................................................aauugcacGguucccggccugcu....................   9       1   seq
......................................................................aauugcacuguucccggccugNu....................   1       1   seq
......................................................................aauugcaUuguucccggccugcu....................   28      1   seq
......................................................................aaAugcacuguucccggccugcu....................   235     1   seq
......................................................................aGuugcacuguucccggccugcu....................   26      1   seq
......................................................................aauugcacuguucUcggccugcu....................   201     1   seq
......................................................................aauCgcacuguucccggccugcu....................   39      1   seq
......................................................................aauugcacuguucccggccugcC....................   59      1   seq
......................................................................aauugcacuNuucccggccugcu....................   1       1   seq
......................................................................aauugcacuguucccggcGugcu....................   27      1   seq
......................................................................aauugcacuguucccgAccugcu....................   45      1   seq
......................................................................aauugcacuguucccggccugAu....................   74      1   seq
......................................................................aauugcacuguucccggccAgcu....................   43      1   seq
......................................................................aauugcacuguuAccggccugcu....................   221     1   seq
......................................................................aauugcacuguucccggccCgcu....................   32      1   seq
......................................................................aauugcacuguucccggccuUcu....................   14      1   seq
......................................................................aauugNacuguucccggccugcu....................   11      1   seq
......................................................................aauugcacuguCcccggccugcu....................   38      1   seq
......................................................................aauugcacuguucGcggccugcu....................   14      1   seq
......................................................................Gauugcacuguucccggccugcu....................   360     1   seq
......................................................................aauugcUcuguucccggccugcu....................   37      1   seq
......................................................................aauugcacugAucccggccugcu....................   104     1   seq
```

```
                                               Star                                    Mature
gagugaccaccgcauggcgucguugaaucggaggguccggauggugcaaguuguuaaaacaaguuggucaaauugcacuguucccggccugcugaucucacucggccgauaac
......................................................................aauugUacuguucccggccugcu....................  33      1    seq
......................................................................aauugcGcuguucccggccugcu....................  95      1    seq
......................................................................Cauugcacuguucccggccugcu....................  4       1    seq
......................................................................aauugcacugCucccggccugcu....................  112     1    seq
......................................................................aauugcacuguuUccggccugcu....................  56      1    seq
......................................................................aauugcacuguucccggUcugcu....................  31      1    seq
......................................................................aauGgcacuguucccggccugcu....................  8       1    seq
......................................................................aauugcacuguucAcggccugcu....................  118     1    seq
......................................................................aauugcacuguucccAgccugcu....................  132     1    seq
......................................................................aauugcacuguucccggcUugcu....................  27      1    seq
......................................................................aauugcacuguucccggccugcug...................  27      0    seq
......................................................................aauugcacuguucccggccugcuU...................  24456   1    seq
......................................................................aauugcacuguucccggccugcuC...................  111     1    seq
......................................................................aauugcacuguucccggccugcuA...................  2790    1    seq
......................................................................aauugcacuguucccggccugcuAa..................  458     1    seq
......................................................................aauugcacuguucccggccugcuga..................  2       0    seq
......................................................................aauugcacuguucccggccugcuCa..................  1       1    seq
......................................................................aauugcacuguucccggccugcuUa..................  8       1    seq
......................................................................aauugcacuguucccggccugcuAau.................  4       1    seq
......................................................................aauugcacuguucccggccugcugaA.................  1       1    seq
......................................................................aauugcacuguucccggccugcuUau.................  2       1    seq
.......................................................................aAugcacuguucccggccu.......................  1       1    seq
.......................................................................auugcacuguucccggccu.......................  41      0    seq
.......................................................................auugcacuguucccggccGg......................  2       1    seq
.......................................................................Nuugcacuguucccggccug......................  1       1    seq
.......................................................................auugcacuguucccggccug......................  16      0    seq
.......................................................................aAugcacuguucccggccugc.....................  1       1    seq
.......................................................................auugcacuguucccggccugc.....................  53      0    seq
.......................................................................Guugcacuguucccggccugc.....................  2       1    seq
.......................................................................Guugcacuguucccggccugcu....................  3       1    seq
.......................................................................auugcacuguucccggccugcu....................  155     0    seq
.......................................................................aAugcacuguucccggccugcu....................  4       1    seq
.......................................................................auugcacAguucccggccugcu....................  1       1    seq
.......................................................................auugcacuAuucccggccugcu....................  1       1    seq
.......................................................................auAgcacuguucccggccugcu....................  1       1    seq
.......................................................................auugcacuguucccggccugcuU...................  100     1    seq
.......................................................................auugcacuguucccggccugcuA...................  21      1    seq
........................................................................uugcacuguucccggccu.......................  1       0    seq
........................................................................uugcacuguuccUggccugcu....................  1       1    seq
........................................................................uugcacuguucccggccugcu....................  83      0    seq
........................................................................uugcacuguucccggccugcug...................  1       0    seq
.........................................................................ugcacuguucccggccugc.....................  2       0    seq
.........................................................................ugcacuguucccggccugcu....................  9       0    seq
.........................................................................ugcacuguucccggccugcuA...................  2       1    seq
..........................................................................gcacuguucccggccugc.....................  9       0    seq
..........................................................................gcacuguucccAgccugc.....................  1       1    seq
..........................................................................gcacuguucccggccugcu....................  30      0    seq
..........................................................................gcacuguuccUggccugcu....................  1       1    seq
..........................................................................gcacuguucccggccugcuU...................  1       1    seq
..........................................................................gcacuguucccggccugcuAa..................  1       1    seq
............................................................................acuguucccggccugcuA...................  1       1    seq
....................................................................................cggccugcugaucucacucggc.......  1       0    seq
```
